# Examining a sentiment algorithm on session patient records in an eating disorder treatment setting: a preliminary study

**DOI:** 10.3389/fpsyt.2024.1275236

**Published:** 2024-03-13

**Authors:** Sophie M. Huisman, Jannis T. Kraiss, Jan Alexander de Vos

**Affiliations:** ^1^ Department of Psychology, Health and Technology, Centre for eHealth and Wellbeing Research, University of Twente, Enschede, Netherlands; ^2^ Department of Research, GGZ Friesland Mental Healthcare Institution, Leeuwarden, Netherlands; ^3^ Human Concern, Centrum Voor Eetstoornissen, Amsterdam, Netherlands

**Keywords:** eating disorders, automated sentiment analysis, session patient records, validation, sentiment extraction

## Abstract

**Background:**

Clinicians collect session therapy notes within patient session records. Session records contain valuable information about patients’ treatment progress. Sentiment analysis is a tool to extract emotional tones and states from text input and could be used to evaluate patients’ sentiment during treatment over time. This preliminary study aims to investigate the validity of automated sentiment analysis on session patient records within an eating disorder (ED) treatment context against the performance of human raters.

**Methods:**

A total of 460 patient session records from eight participants diagnosed with an ED were evaluated on their overall sentiment by an automated sentiment analysis and two human raters separately. The inter-rater agreement (IRR) between the automated analysis and human raters and IRR among the human raters was analyzed by calculating the intra-class correlation (ICC) under a continuous interpretation and weighted Cohen’s kappa under a categorical interpretation. Furthermore, differences regarding positive and negative matches between the human raters and the automated analysis were examined in closer detail.

**Results:**

The ICC showed a moderate automated-human agreement (ICC = 0.55), and the weighted Cohen’s kappa showed a fair automated-human (k = 0.29) and substantial human-human agreement (k = 0.68) for the evaluation of overall sentiment. Furthermore, the automated analysis lacked words specific to an ED context.

**Discussion/conclusion:**

The automated sentiment analysis performed worse in discerning sentiment from session patient records compared to human raters and cannot be used within practice in its current state if the benchmark is considered adequate enough. Nevertheless, the automated sentiment analysis does show potential in extracting sentiment from session records. The automated analysis should be further developed by including context-specific ED words, and a more solid benchmark, such as patients’ own mood, should be established to compare the performance of the automated analysis to.

## 1 Introduction

Eating disorders (EDs) are serious psychological disorders characterized by disturbed eating patterns that can lead to (severe) somatic and psychological complications (1, 2). The three most common EDs are anorexia nervosa (AN), bulimia nervosa (BN), and binge-eating disorder (BED) (1). EDs that do not meet the criteria of one of the aforementioned disorders but do create significant distress or functional impairment are classified under the category of “other specified feeding and eating disorders” (OSFED) (2). The lifetime prevalence of all EDs is 8.4% for women and 2.2% for men, which has increased during the last decades (3–5).

Despite the different types of therapy available for EDs, they remain challenging to treat and are followed by high levels of relapse, reflecting the often chronic nature of these disorders (6–9). Hence, it is essential to better understand and monitor the recovery process to protect individuals against relapse. One way to facilitate recovery is by monitoring the responsiveness of patients to treatment with routine outcome monitoring (ROM) (10). The ROM is an instrument to periodically evaluate patients’ progress through using diagnostic indicators and severity scales (11, 12). ROM can alert therapists when treatment is ineffective, indicate a worsening of symptoms, or reassure patients by providing insight into slight improvements in their situation (13).

However, ROM requires patients to fill out self-report questionnaires, which may lead to subjective bias resulting in an over- or underestimation of patient’s states (14). Furthermore, ROM is supposed to be administered at fixed time intervals during treatment, which is burdensome for patients and time-consuming for therapists, making it costly and not always feasible within clinical settings (11, 15–17). As a result, ROM is often only completed at the beginning and end of therapy, leading to a limited representation of patients’ treatment progress (16, 18). The limitations of the ROM demonstrate that therapists could benefit from a less burdensome procedure and data utilization to continuously monitor patients’ treatment progress.

Therapists already collect information about patients’ treatment progress within session-by-session patient records (session records) (19). Clinicians write the session records after therapy sessions that may contain valuable information, such as patients’ reactivity to and states during treatment, details of therapeutic conversations, and clinicians’ impressions of the patient (20, 21). Session records are essential to treatment as they improve patient care by ensuring effective communication between clinicians and can support the substantiation of treatment choices (22, 23). Exhaustive evaluation of the session records could yield insightful information into patients’ treatment process and progress.

However, the utilization of session records in research is limited due to the records being lengthy and complex, requiring more advanced and customized approaches to manage the difficulties in extracting information from such texts (21, 24–26). The session records are classified as unstructured data, meaning that the qualitative texts are not stored in an organized predefined format, making them challenging to analyze with conventional analysis techniques (27, 28). One conventional method to analyze such texts is by using human raters. However, this task is demanding and time-consuming and is often not feasible when large amounts of text data are involved (29). Throughout the last few years, new techniques have emerged that allow for more cost-effective and efficient analysis of unstructured text data (30). One such method is natural language processing (NLP) in which computer programs attain the ability to understand natural language in text or spoken words (31). A subfield within NLP is automated sentiment analysis, aiming to analyze natural language by using an algorithm operating through a set of rules to identify sentiment encompassing attitudes, emotions, appraisals, and the emotional tone within a text (32). Hence, automated sentiment analysis could be particularly suited to analyze session records because these often contain sentiment.

Sentiment analysis has become increasingly popular and was mainly used for the mining of sentiment from online customer reviews. However, prior research has started to examine the sentiment of patients’ medical records, which showed potential regarding the mining of sentiment from such texts (33–36). Despite this, sentiment analysis applications within clinical practice remain limited; especially, the sentiment within session records has hardly been examined.

A few sentiment analysis studies have been executed within a clinical setting. A study by Provoost et al. (37) investigated the performance of an automated sentiment analysis on texts from online behavioral therapy interventions regarding different psychological disorders against a set of human raters. They found that the sentiment analysis performed similarly to the human raters in discerning sentiment from such mental health texts. Furthermore, a study investigating the performance of four different sentiment analyses on healthcare-related texts against a human baseline found three sentiment analyses to have fair agreement and one to have moderate agreement with the human raters (38). Moreover, a study evaluating the sentiment on videos and comments about AN found a fair agreement between the automated sentiment analysis and human raters (39). However, to date, only one study has investigated the performance of an automated sentiment analysis on written statements from patients diagnosed with anorexia nervosa regarding their body perception (40). This study showed that a relationship existed between patients’ vocabulary in written texts and their mental states, Furthermore, the texts could be categorized in one of the six predefined subcategories related to AN (40).

Despite these studies showing promising results, a challenge within this type of research is that there is no solid benchmark to compare the performance of automated sentiment analyses with, because research regarding the analysis of sentiment from session records within the mental healthcare domain is very limited. For example, Provoost and colleagues (37) used the agreement among the human raters as benchmark to compare the performance of the automated sentiment analysis too. Their research suggested that the automated sentiment analysis performed similar to the human raters. However, the aforementioned study showed a moderate human-human agreement, meaning that the human raters differed in many cases regarding the sentiment of the texts. Hence, because of a lack of consensus between raters, it cannot be determined with certainty whether the performance of the automated sentiment analysis is either “good” or “bad”. Another point is that this research is conducted within the field of clinical psychology; therefore, thorough research is required on new technologies before they can actually be applied in practice (37, 41). Furthermore, automated sentiment analyses can be highly context-specific, as texts within different contexts may require different vocabulary and language, such as analyzing social media texts in contrast to clinical documents (42–45). Thus, the vocabulary within an ED context may differ from the vocabulary used within other domains of mental healthcare.

In all, limited evidence exists on the performance of automated sentiment analyses on session patient records within an ED treatment context. The automated sentiment analysis is not tailored to an ED context; however, because of the context specificity of such analyses, it is not clear whether an automated sentiment analysis (without tailoring) can extract sentiment reliably and validly from session records within such a context. Furthermore, because of little understanding about the application of an automated sentiment analyses within clinical practice, it must be thoroughly researched and validated before such analyses can be applied within the clinical field. The session records are readily available to examine patients’ treatment progress; therefore, efficient analysis of these records by an automated sentiment analysis may provide a less burdensome method for both patients and clinicians to monitor treatment progression over time and be used on different texts related to EDs. Therefore, this study will examine how an existing Dutch automated sentiment analysis evaluates unstructured text data from session patient records compared to human raters.

## 2 Materials and methods

### 2.1 Participants

Participants were Dutch patients with the criteria of having a minimum age of 17 at the time of providing an informed consent and an ED diagnosis during data collection. A total of 149 patients were asked to sign the consent form, of which 12.1% rejected. A total of 131 patients provided consent. A random selection was made for this preliminary study, including patients with different ED diagnoses and a minimum of forty session records.

The sample consisted of eight patients: two patients diagnosed with AN, three patients with BN, one with BED, and two with OSFED. Five patients were between the ages of 21 and 25, two between the ages of 26 and 30, and one between the ages of 31 and 35. The average duration of patients’ treatment up to the start of the study was approximately 10 months (SD = 4.8).

### 2.2 Procedure

Patients’ session records were evaluated on their sentiment by an automated sentiment analysis and separately by two human raters. The two human raters examined each session patient record and allocated a sentiment score to each record individually.

Data collection occurred between February 2019 and April 2022, during which participants received outpatient treatment at a specialized ED treatment institution in The Netherlands (46). Patients were diagnosed with an ED by a psychiatrist or clinical psychologist in collaboration with an intake team. Participants visited their therapist once or twice a week for individual face-to-face treatment sessions, which were partly online due to the restrictions regarding the COVID-19 pandemic in The Netherlands (47). Therapy sessions concerned topics regarding recovery, autonomy, and decreasing problematic eating behavior using cognitive behavioral therapy and insight-giving therapy. Patients also received homework after the sessions to apply what they had learned (46). Furthermore, at the start of treatment, each patient received an account for an eHealth environment in which questionnaires and exercises were offered, where patients were provided with a brochure explaining the aim of the research as well. Patients were able to contact the researchers for further information and signed an informed consent form which they could withdraw from when they no longer wished to participate (see **Appendix A** and **B**).

The client advisory board of Human Concern advised on the execution of the study regarding adherence to ethical principles concerning patient privacy, possible risk, and harm and clarity of the study brochure. The study protocol was approved by the board of directors at Human Concern and the Ethical Committee of the University of Twente (EC-220422).

### 2.3 Materials

#### 2.3.1 Session patient record data

The data utilized for this study were session patient record data. The session records were written electronically within the used system by the clinicians during treatment; they were free to use their own format in writing the records and could include any information they deemed important. The records included information from therapy sessions, treatment progression, ROM results, and patients’ background information. The records varied in length, language, and format. However, not all session records were suited for the analysis. Some records only contained brief information about arranged appointments with other clinicians or institutions or descriptions of actions taken by the clinician(s) regarding administrative activities. Therefore, records that included one (or several) of the aforementioned actions or contained less than five words were excluded from the analysis by the human raters. In contrast, the automated analysis only excluded records with less than five words or records that did not include sentiment words.

#### 2.3.2 Anonymization

The model “deduce” tailored to the Dutch language was executed on the pseudonymized session patient records to anonymize the data (48). First, patient and postal codes, addresses, email addresses, telephone numbers, URLs, and other contact information, including those of relatives, clinicians, and other care providers and institutions, were excluded. Second, the session records were tokenized; names and initials were changed to (NAME-1) and dates to (DATE-1); and dates indicating the start or end of treatment were transformed to a month and year, ages to (AGE), and locations or cities to (LOCATION-1).

#### 2.3.3 Automated sentiment analysis

To analyze the sentiment within the session records, an automated sentiment analysis from 6Gorillas tailored to the Dutch language and mental healthcare domain was used (49). Before analyzing the data, the sentiment analysis automatically pre-processed the data by transforming capital letters to lowercase letters and removing stop words, numbers, words with only one character, and underscores to improve the data mining functionality and prevent misleading results (50). The automated sentiment analysis employed a top-down lexicon-based approach, using three lexicons to extract sentiment. The primary lexicon used was from NRC Word-Emotion Association containing English sentiment words translated into Dutch; furthermore, a healthcare-specific lexicon created by 6Gorillas and an adjustment dictionary from Ynformed (a data science company) changed or removed words with multiple meanings within a text (51).

The lexicon indicated whether a positive or negative sentiment score was awarded to a sentiment-bearing word within a session record. Furthermore, the automated sentiment analysis searched for words prior to a sentiment-bearing word to examine the semantic context by using N-grams, including bigrams (a two-word sequence) and trigrams (a three-word sequence). Consequently, the automated analysis could account for negations that reverse the polarity of a sentence (e.g., “not good”) and strengthening words (“extremely good”) (52, 53). The sentiment score of a bigram was calculated by scoring the sentiment-bearing word with either “0,” “+1,” or “−1,” which was multiplied by two when the preceding word was a reinforcer, and the sentiment score was inverted when the preceding word was a negation. The final score was calculated by adding all the bigram scores of a session record divided by the total number of bigrams (49). For trigrams, the same approach was used; the sentiment-bearing word determined the sentiment, and the two preceding words indicated whether the score was inverted or reinforced. The final score was calculated by adding all the trigrams scores of a session record divided by the total number of trigrams.

A final overall sentiment score was awarded to each session record, which was an average of all the sentiment scores within a record ranging between an interval of −1 and 1. Higher (positive) scores indicated greater positive sentiment, scores close to zero indicated a neutral sentiment, and lower (negative) scores indicated a greater negative sentiment of the record.

#### 2.3.4 Human sentiment analysis

The procedure of Provoost and colleagues (37) was followed for the human sentiment analysis as a guideline because this was the only study examining the extraction of sentiment from texts within a Dutch mental health context.

Two human raters were involved in the human sentiment analysis; the first author was considered the first human rater, and the last author the second human rater. First, the human raters rated the first 20 session records together to explore variations in their ratings. After individually rating a session record, they discussed their reasoning and justifications for their scores. This collaborative approach served as the foundation for the preliminary protocol. Subsequently, they independently rated the next eighty session records. After, a feedback session was arranged to discuss issues and difficulties concerning the sentiment rating, upon which the protocol was refined and finalized. Hereafter, the new protocol was used to evaluate the overall sentiment of the remaining session (see **Appendix C**). Every record was rated on a scale from 1 to 7, with “1” indicating very negative, “2” indicating negative, “3” indicating somewhat negative, “4” indicating neutral, “5” indicating somewhat positive, “6” indicating positive, and “7” indicating very positive.

The category “neutral” was assigned when a record was considered objective (including no sentiment) or contained about the same number of positive and negative sentiments. Furthermore, a separate category “mixed” was created to indicate that a session record contained both an equal number of positive and negative sentiment. Because the automated sentiment analysis frequently scored such records as “neutral,” the category “mixed” was created to explore the frequency of this occurrence.

### 2.4 Data analysis

Analyses were performed within the statistical program R (54) and Statistical Package of the Social Sciences (SPSS) 28 (55). The alpha level was set at 0.05.

#### 2.4.1 Data preparation

The raw sentiment scores from the automated sentiment analysis and scores from the human raters were standardized in order to compare the automated and human sentiment analysis.

##### 2.4.1.1 Automated sentiment analysis

Categories were created for the standardized sentiment scores on the session records from the automated analysis. For the standardized sentiment scores, no score of zero existed indicating the category “neutral,” given the wide range of scores generated by the automated analysis. Therefore, the category “neutral” was defined as a range bounded by the first positive and first negative standardized sentiment score. The category “negative” was defined by the scores below the first negative standardized sentiment score, and the category “positive” was defined by the scores above first positive standardized sentiment scores. Consequently, the categories for the standardized overall sentiment scores from the automated analysis were defined as follows: negative for values smaller than −0.03 and positive for values larger than 0.11.

##### 2.4.1.2 Human sentiment analysis

Categories were created for the raw sentiment scores of each human rater as these are similar to the standardized sentiment scores. Values smaller than 4 were categorized as negative, values larger than 4 as positive, and scores equal to 4 as neutral.

Furthermore, the sentiment scores of each human rater were standardized. An overall human sentiment score was calculated by taking the average of both raters’ sentiment score on each record, which was standardized and is referred to as the average human rating. A contingency table was created, including both human raters’ raw sentiment scores and a frequency distribution of negative, neutral, and positive scores between the human raters.

#### 2.4.2 Human-automated agreement

##### 2.4.2.1 Categorical interpretation

A weighted Cohen’s kappa was calculated to assess the inter-rater agreement (IRR), which measured the extent that two (or more) examiners agreed on their assessment decisions (56). The weighted Cohen’s kappa accounted for ordinal categorical data and was used to measure a text’s polarity in terms of its direction (category). The weighted Cohen’s kappa was calculated to examine the IRR between the standardized categorical sentiment scores of the automated analysis and categorical scores of rater 1 and rater 2 (57, 58). Values for the weighted Cohen’s kappa range between −1 and 1; the degree of agreement was interpreted as none (<0), slight (0 to 0.20), fair (0.21 to 0.4), moderate (0.41 to 0.60), substantial (0.61 to 0.80), or almost perfect reliability (> 0.80) (59).

##### 2.4.2.2 Continuous interpretation

The intra-class correlation (ICC) can be used to assess the IRR on continuous data and data with missing values (58). The ICC correlated the standardized sentiment scores of the automated analysis against the standardized sentiment scores of rater 1 and rater 2 to measure the intensity of the agreement between the two analyses, accounting for a two-way mixed effect model based on an absolute agreement (60). Values for the ICC ranged between 0 and 1; the degree of agreement was interpreted as poor (<0.50), moderate (0.50 to 0.75), good (0.75 to 0.90), and excellent reliability (>0.90) (60).

#### 2.4.3 Human-human agreement

##### 2.4.3.1 Categorical interpretation

A weighted Cohen’s kappa was calculated to assess the IRR between the categorical scores of the human raters. The Cohen’s kappa was interpreted as aforementioned. 

##### 2.4.3.2 Continuous interpretations

The ICC was calculated to assess the IRR between the raw sentiment scores of the human raters. The ICC was interpreted as aforementioned.

#### 2.4.4 Human-automatic agreement per individual patient

##### 2.4.4.1 Continuous interpretation

The ICC was calculated to assess the IRR between the standardized scores of the automated sentiment analysis and each human rater for each patient individually. The ICC was interpreted as aforementioned.

##### 2.4.4.2 Differences between the automated sentiment analysis and human sentiment analysis

A line graph was created for each patient to visualize the differences between the automated and human sentiment analysis, illustrating a patient’s sentiment score over time. The graphs included the standardized automated sentiment analysis’s and average human sentiment scores on each session record (y-axis) and the number of records (x-axis). The average human sentiment rating was used due to the good (ICC = 0.89) and substantial (k = 0.68) human-human agreement. Furthermore, deviations in sentiment scores between the automated and human raters were examined and reflected upon. The sentiment-bearing words and its assigned positive or negative match by the automated sentiment analysis and human raters were explored in closer detail. Accordingly, a word list was created for words specific to an ED context, which were not considered during the automated analysis. Furthermore, a word list was created for words considered of positive or negative sentiment by the automated analysis, which were not considered or considered of the opposite sentiment by the human raters.

## 3 Results

### 3.1 Patient session records

Out of the total 460 session patient records with an average of 57.50 (SD = 48.02) records per patient, 268 (58.3%) records were deemed relevant for the analysis by the first human rater and 263 (57.1%) by the second rater, whereas the automated analysis scored 315 (68.5%) records as relevant for the analysis.

### 3.2 Categorical comparison between the human raters and automated sentiment analysis

The automated sentiment analysis rated more session records as positive compared to the human raters, whereas the scores for the categories neutral and negative from the automated analysis and human raters are closer to each other (see **Table 1**). The human raters showed similar ratings for each category, with the largest difference for the category “positive” (see **Table 1**).

**Table 1:** Comparison of categorical sentiment evaluations on the session patient records from the human raters and automated sentiment analysis.

	Rater 1 *N* (%)	Rater 2 *N* (%)	Automated Analysis *N* (%)
Negative (%)	126 (47.0%)	127 (48.3%)	135 (36.8%)
Neutral (%)	64 (23.9%)	70 (26.6%)	64 (20.3%)
Positive (%)	78 (29.1%)	66 (25.1%)	116 (42.9%)
Total	268	263	315

Furthermore, the human raters showed the most consensus on the scoring of the session records in the “positive” category, followed by the “negative” category (see **Table 2**). The lowest consensus was observed for the category “neutral” where, when one human rater categorized a record as “neutral,” the other human rater more often categorized the record in one of the other two categories.

**Table 2:** Comparison between the human raters’ categorical sentiment evaluations on the patient session records.

	Rater 2				
Rater 1		Negative *N* (%)	Neutral N (%)	Positive N (%)	Total N (%)
	Negative	106 (83.5%)	14 (20.0%)	5 (7.6%)	125 (47.5%)
	Neutral	14 (11.0%)	43 (61.4%)	4 (6.1%)	61 (23.2%)
	Positive	7 (5.5%)	13 (18.6%)	57 (86.4%)	77 (29.3%)
	Total	127	70	66	263

### 3.3 Automated-human agreement

#### 3.3.1 Categorical interpretation

The weighted Cohen’s kappa indicated a fair agreement, k = 0.29 (95% CI, 0.199 to 0.387, p < 0.001), between the automated sentiment analysis and rater 1 regarding overall sentiment of the session records.

The weighted Cohen’s kappa indicated a fair agreement, k = 0.29 (95% CI, 0.191 to 0.378, p < 0.001), between the automated sentiment analysis and rater 2 regarding overall sentiment of the session records.

#### 3.3.2 Continuous interpretation

The ICC analysis revealed a moderate IRR [ICC = 0.51, CI = 0.37–0.61, F(267, 267) = 2.02, p < 0.001] between the automated analysis and rater 1 regarding overall sentiment on the session records.

The ICC analysis revealed a moderate IRR [ICC = 0.57, CI = 0.43–0.65, F(262, 262) = 2.245 p < 0.001] between the automated analysis and rater 2 regarding overall sentiment on the session records.

### 3.4 Human-human agreement

#### 3.4.1 Categorical interpretation

The weighted Cohen’s kappa indicated a substantial agreement [k = 0.68 (95% CI, 0.62 to 0.75), p = 0.000] between rater 1 and rater 2 regarding overall sentiment on the session records.

#### 3.4.2 Continuous interpretation

The ICC analysis revealed a good IRR [ICC = 0.89, CI = 0.86–0.91, F(262, 262) = 9.02, p < 0.001] between rater 1 and rater 2 regarding overall sentiment on the session records.

### 3.5 Automated-human agreement per individual patient

#### 3.5.1 Continuous interpretation

The ICC revealed a poor IRR for participants 1 (OFSED), 4 (AN), and 6 (BN) for rater 1 (see **Table 3**). The ICC revealed a poor IRR for participants 1, 4, and 5 (BED) for rater 2 (see **Table 4**). Moderate ICC values were found for the remaining participants for both raters. The values were significant for four cases for rater 1 and five cases for rater 2 (see **Tables 3**, **4**).

**Table 3:** Intra-class correlation value for the agreement between the first human rater and the automated sentiment analysis per participant.

	ICC	95% CI		F-statistics		
		Lower	Upper	Value	df1	df2
Participant 1 (OFSED)	0.13	−0.47	0.48	1.14	56	56
Participant 2 (AN)	0.63	0.34	0.79	2.65**	49	49
Participant 3 (BN)	0.50	−0.29	0.82	2.10	15	15
Participant 4 (AN)	0.37	−0.18	0.67	1.58	41	41
Participant 5 (BED)	0.60	0.25	0.78	2.42**	43	43
Participant 6 (BN)	0.38	−0.60	0.75	1.57	20	20
Participant 7 (BN)	0.69	0.19	0.87	3.05*	19	19
Participant 8 (OFSED)	0.60	−0.11	0.85	2.41*	17	17

ICC, intra-class correlation; CI, confidence intervals, * < 0.05, ** < 0.01.

**Table 4:** Intra-class correlation value for the agreement between the second human rater and the automated sentiment analysis per participant.

	ICC	95% CI		F-statistics		
		Lower	Upper	Value	df1	df2
Participant 1 (OFSED)	0.30	−0.18	0.59	1.44	55	55
Participant 2 (AN)	0.72	0.51	0.84	3.52**	49	49
Participant 3 (BN)	0.66	−0.05	0.88	2.93*	15	15
Participant 4 (AN)	0.41	−0.09	0.68	1.69*	41	41
Participant 5 (BED)	0.40	0.10	0.67	1.66*	43	43
Participant 6 (BN)	0.69	0.21	0.88	3.24*	18	18
Participant 7 (BN)	0.68	0.14	0.88	3.07*	17	17
Participant 8 (OFSED)	0.54	−0.26	0.83	2.12	17	17

ICC, intra-class correlation; CI, confidence intervals, * < 0.05, ** < 0.01.

#### 3.5.2 Differences between the automated and human sentiment analysis

**Figure 1:**
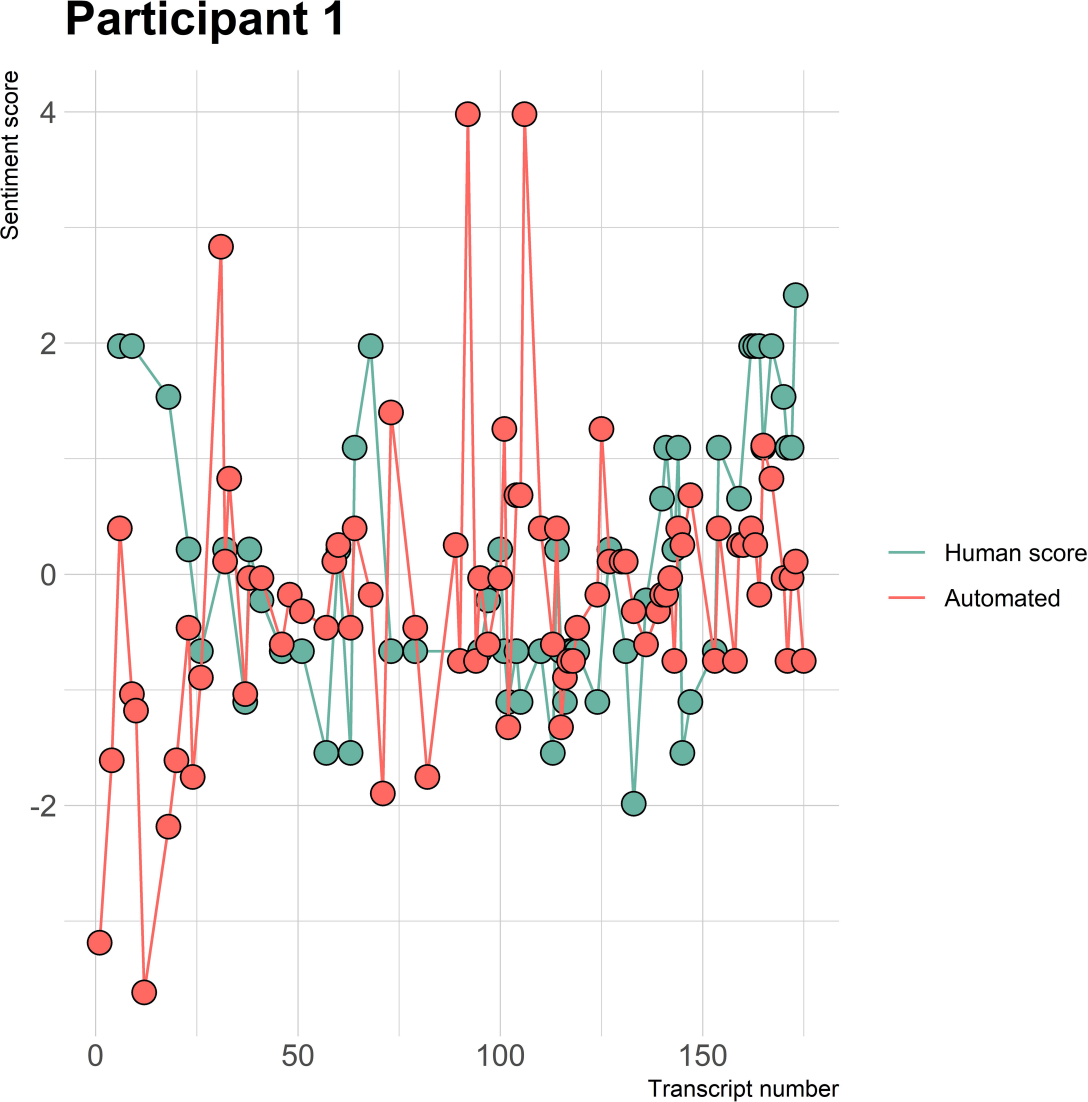
Sentiment scores from the automated sentiment analysis and the human sentiment analysis over time for participant 1 (OFSED) (N = 175).

**Figure 2:**
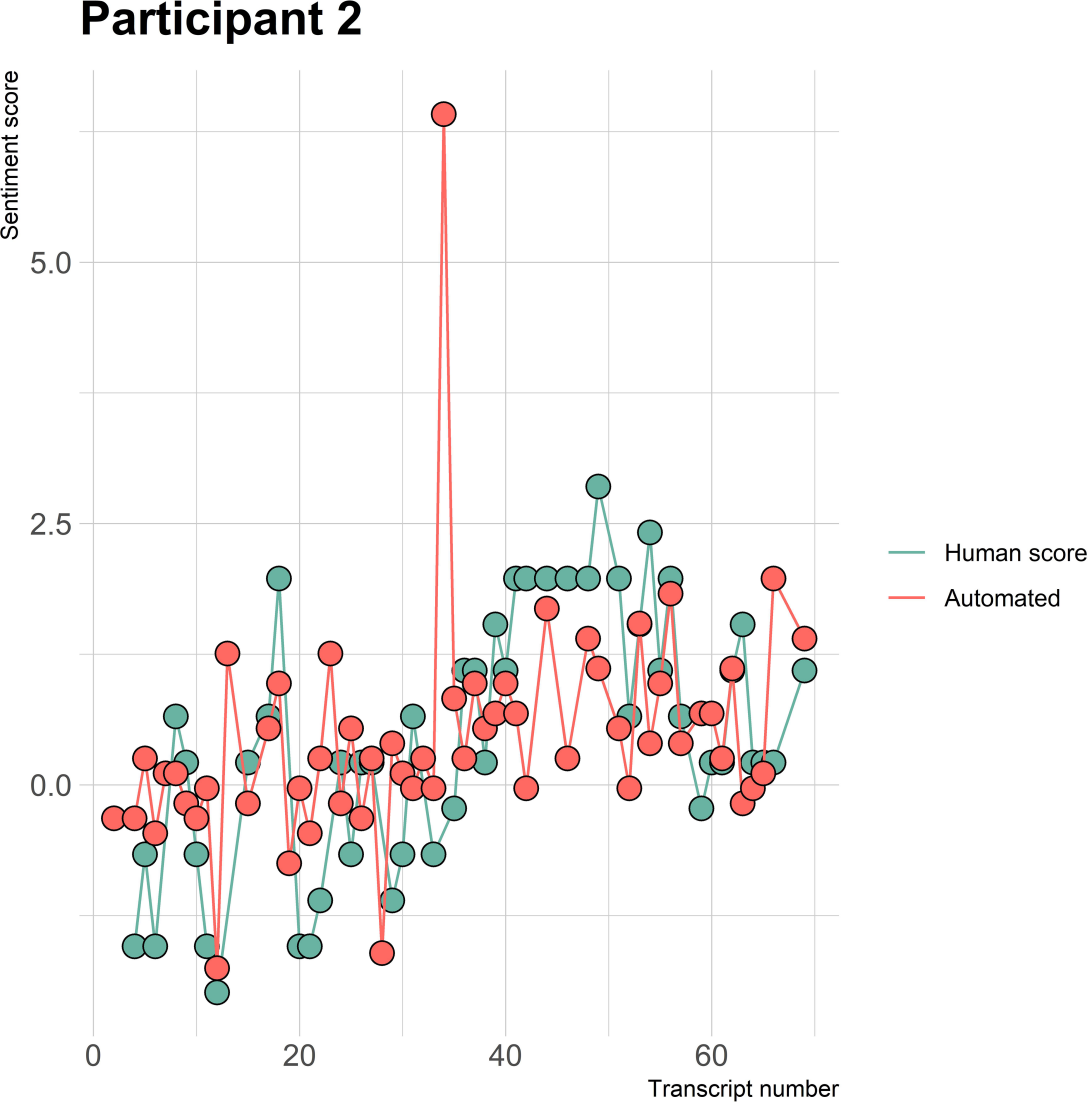
Sentiment scores from the automated sentiment analysis and the human sentiment analysis over time for participant 2 (AN) (N = 68).

**Figure 3:**
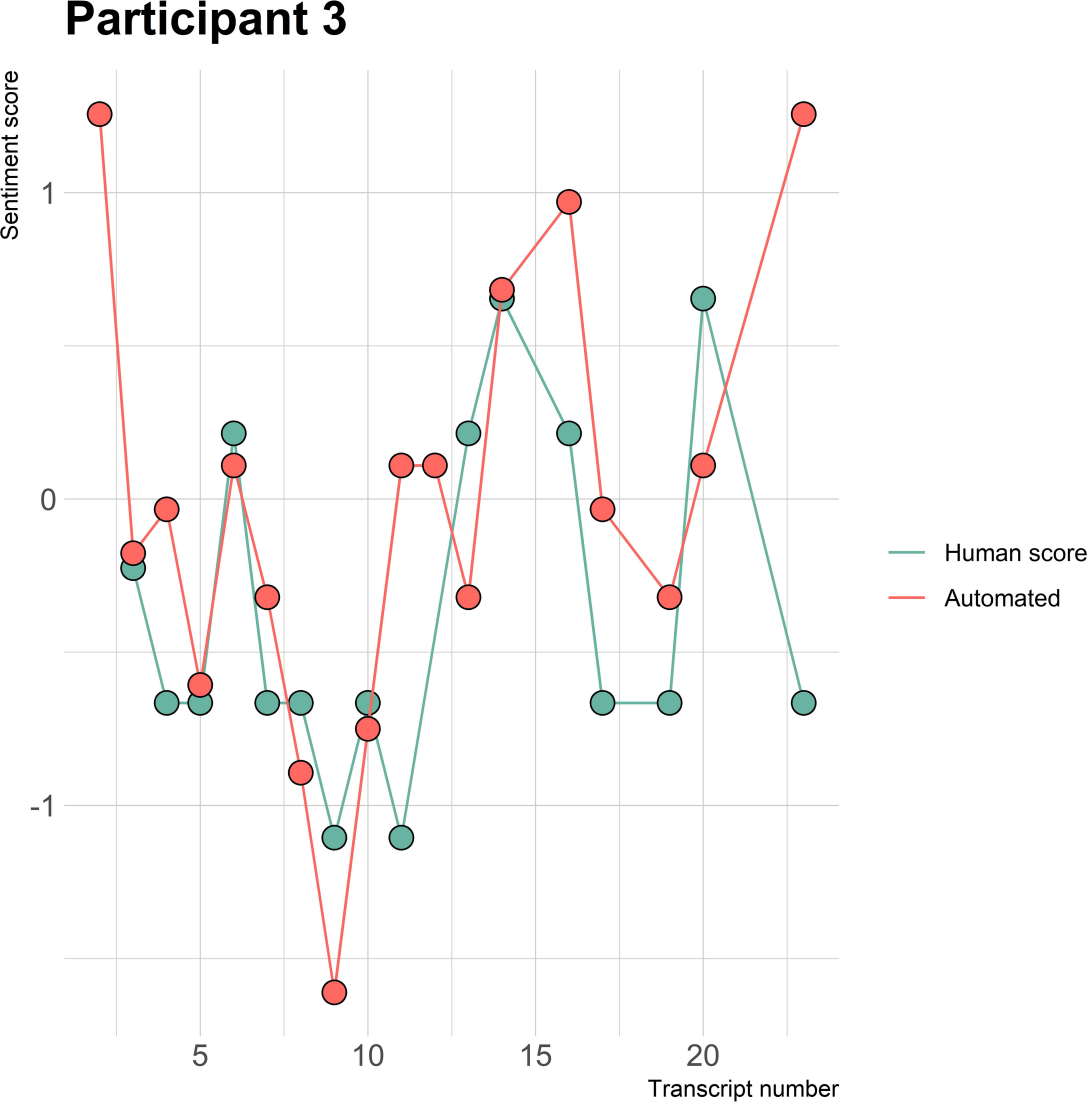
Sentiment scores from the automated sentiment analysis and the human sentiment analysis over time for participant 3 (BN) (N = 22).

**Figure 4:**
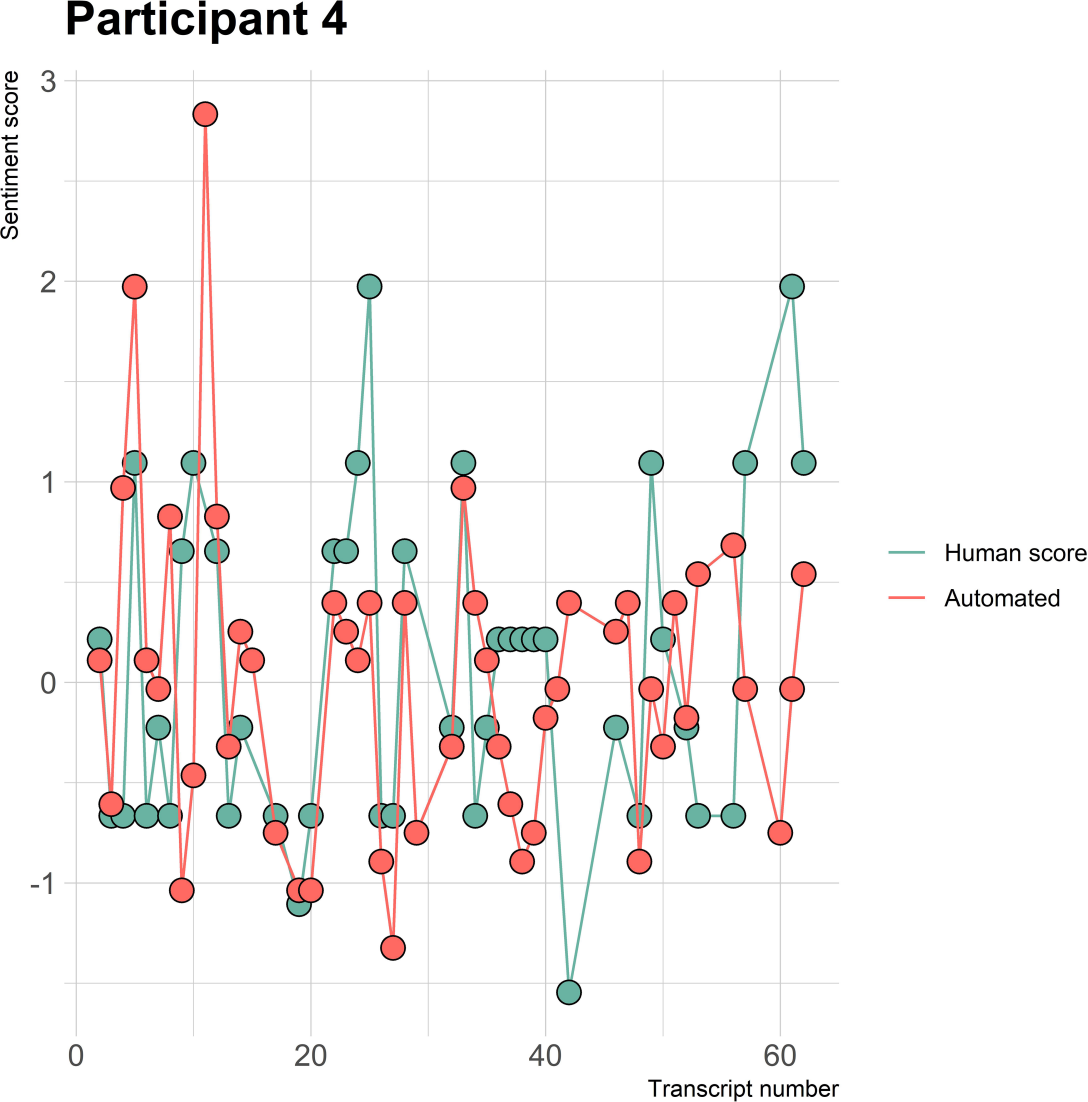
Sentiment scores from the automated sentiment analysis and the human sentiment analysis over time for participant 4 (AN) (N = 61).

**Figure 5:**
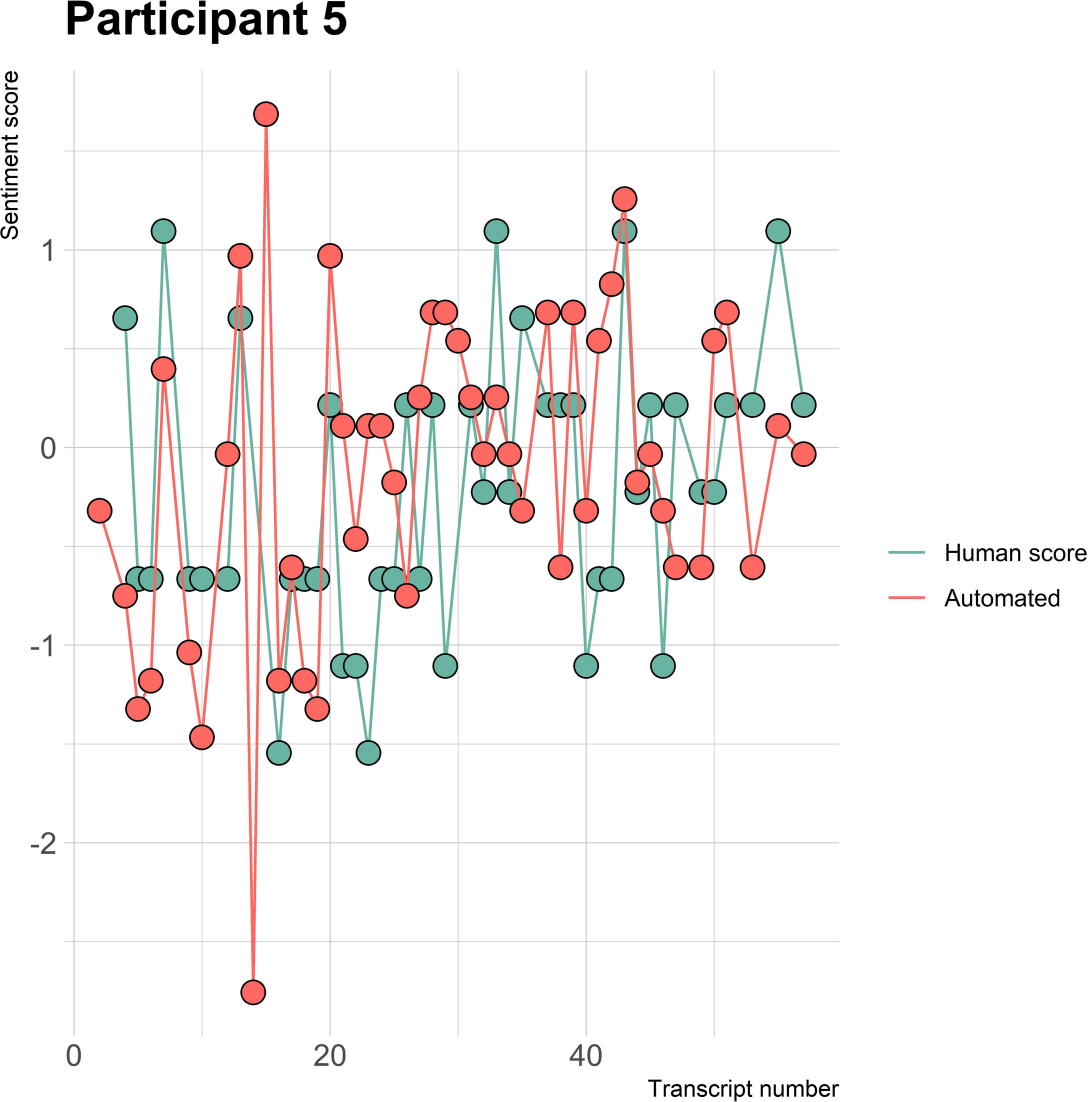
Sentiment scores from the automated sentiment analysis and the human sentiment analysis over time for participant 5 (BED) (N = 56).

**Figure 6:**
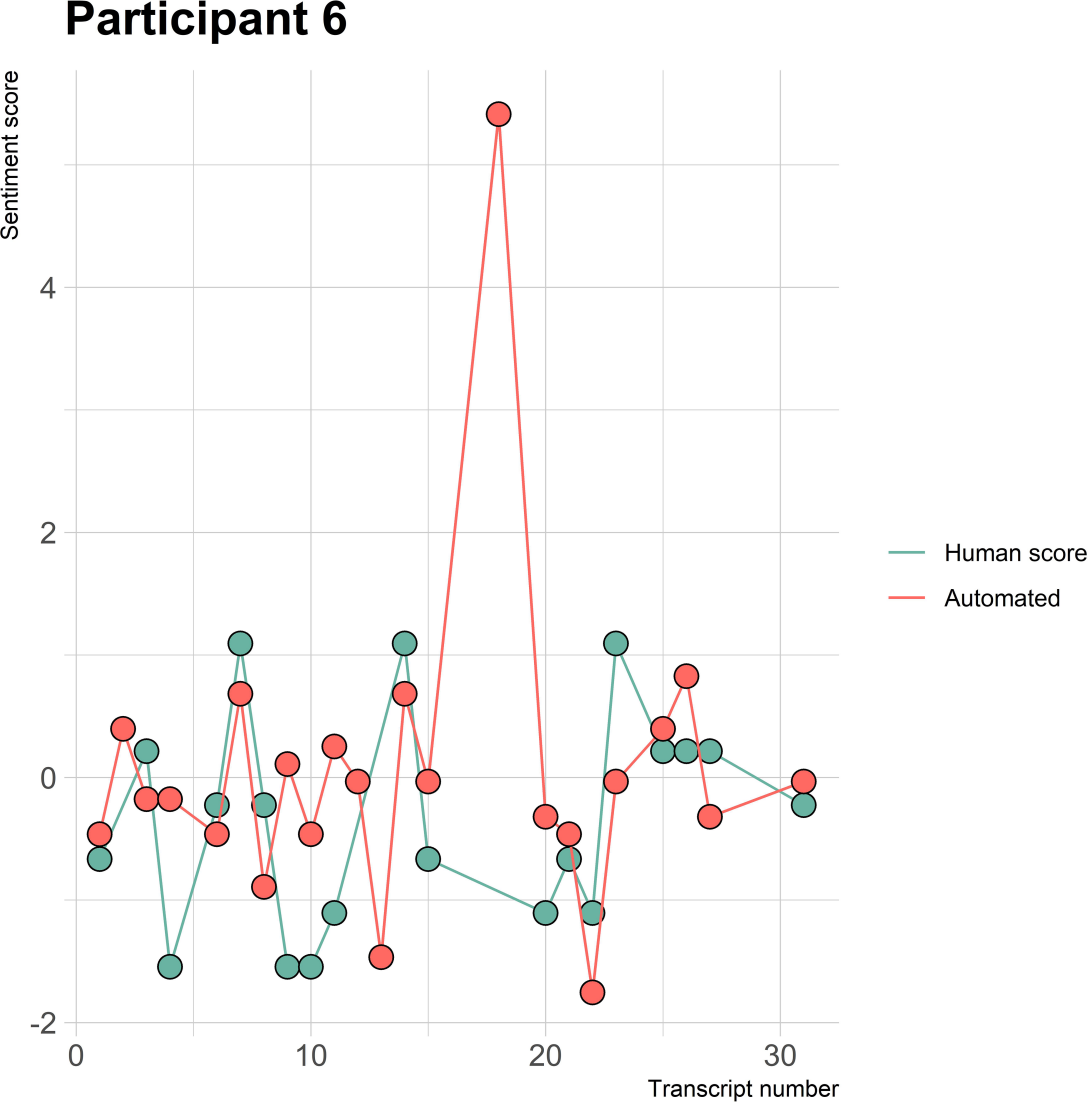
Sentiment scores from the automated sentiment analysis and the human sentiment analysis over time for participant 6 (BN) (N = 30).

**Figure 7:**
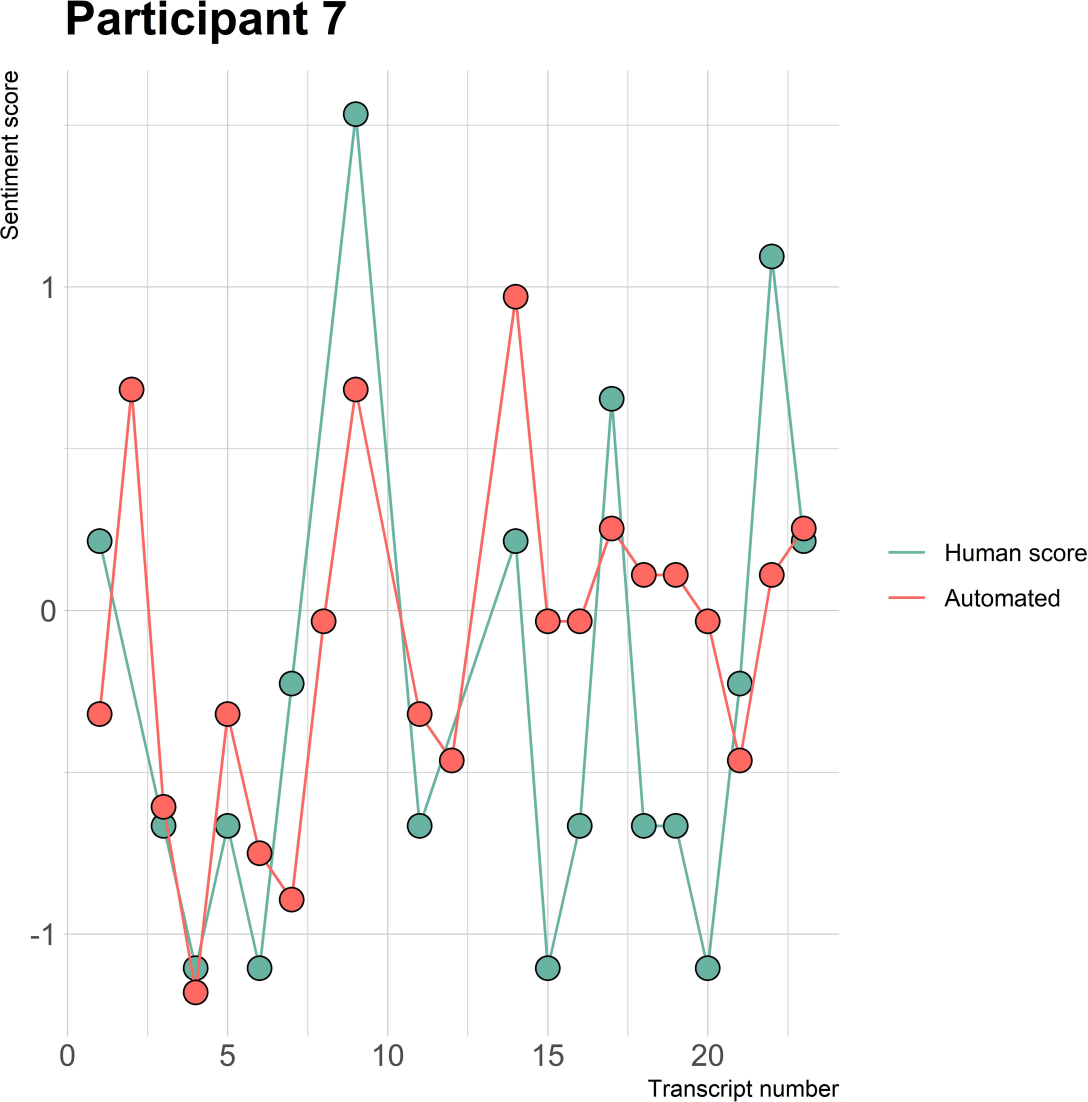
Sentiment scores from the automated sentiment analysis and the human sentiment analysis over time for participant 7 (BN) (N = 22).

**Figure 8:**
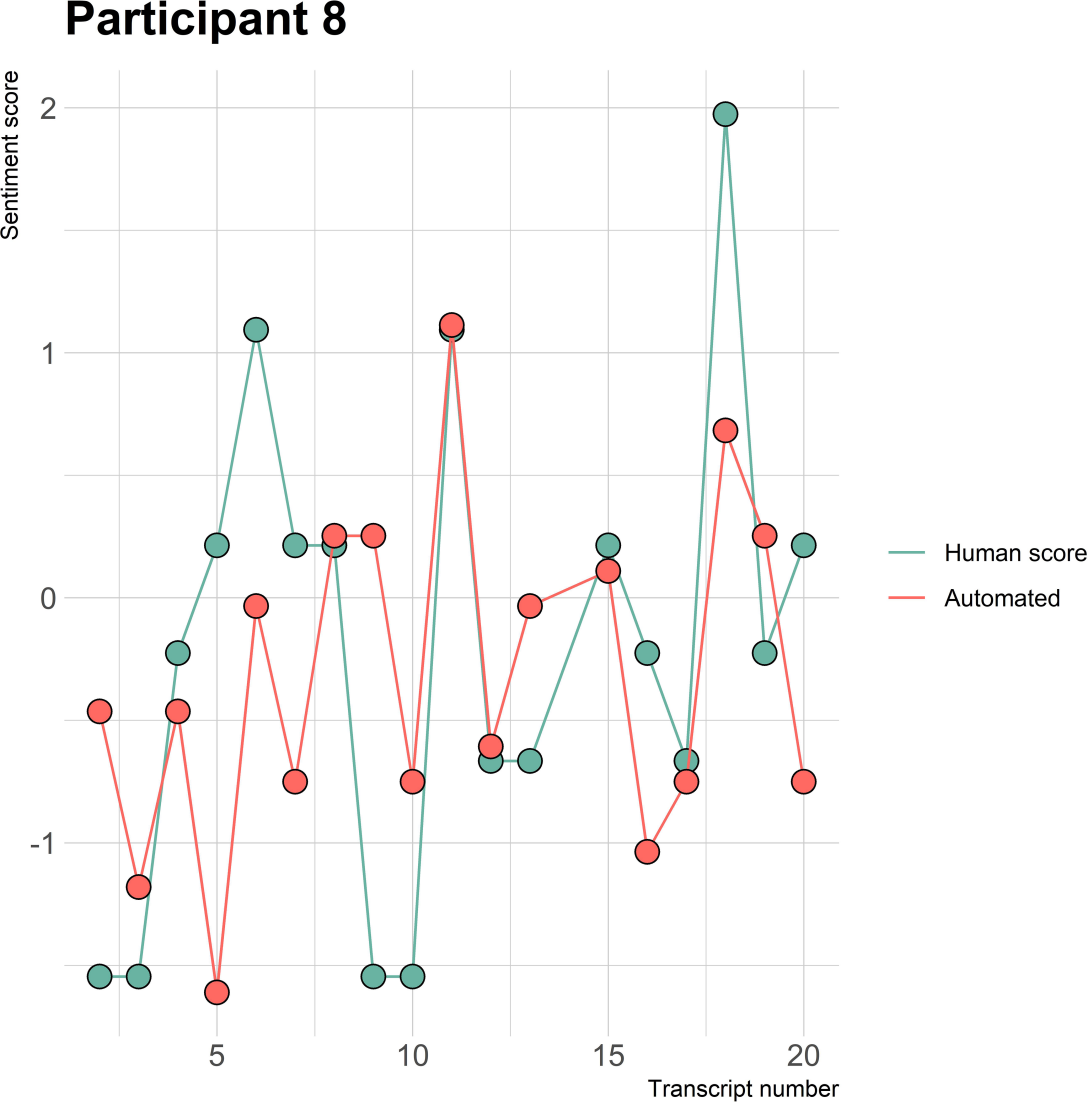
Sentiment scores from the automated sentiment analysis and the human sentiment analysis over time for participant 8 (OFSED) (N = 19).

The visualizations of the sentiment over time per patient regarding sentiment scores from the automated analysis and human raters can be seen in **Figures 1**–**8**. **Figure 1** shows a large difference between the average human rating and the automated analysis on session record 106 of participant 1, where the automated analysis showed a sentiment score of 4.0; however, the human raters identified this record as irrelevant. Likewise, in **Figure 2**, the automated sentiment analysis peaked at record 34 of participant 2, whereas the human raters considered this record irrelevant. Participants 4, 5, and 6 illustrate this occurrence as well, showing a larger peak of the automated analysis without the human raters having assigned a sentiment score to the record in question, such as on record, 10, 13, and 17, respectively, in **Figures 4**-**6**. The automated sentiment analysis presenting a considerably larger sentiment score compared to the human rater is often paired with the human raters evaluating the session record as irrelevant.

##### 3.5.2.1 Sentiment words specific to ED context.

The automated sentiment analysis did not consider words specific to an ED context. An example can be seen from participant 4 diagnosed with AN in session record 37, where the average human rating showed a sentiment score of 1.97 and the automated sentiment analysis a score of 0.40 (see **Figure 4**). When examining the positive and negative matches from the automated analysis on the record, it was observed that the automated analysis did not rate certain context-specific positive ED words or expressions. For instance, the automated analysis did not rate the expression “beautiful recovery line,” “feeling more,” or “taking space,” which are of positive sentiment within the context of EDs. The aforementioned examples are not the only ones encountered when examining the differences between positive and negative matches of the human raters and the automated sentiment analysis. Therefore, a list with context-specific ED words and the different diagnoses can be found in **Appendix D**.

Lastly, the automated sentiment analysis categorized certain words to have a positive or negative polarity, which were not considered or considered of the opposite sentiment within the human analysis. For example, the automated analysis indicated “exercising” or “compensating” as a positive match on a record with a patient diagnosed with AN when, in fact, these expressions are mostly not of a positive polarity within such a context. Moreover, the words “emotion regulation” and “body experience” were categorized as a negative match. However, these were not considered sentiment-bearing words in the human analysis. Further differences regarding the positive and negative matches between the automated analysis and human raters can be found in **Appendix E**.

## 4 Discussion

The aim of this study was to examine the performance of an automated sentiment analysis at extracting sentiment from session patient records within an ED treatment context compared to human raters. In addition, the purpose of this study was to provide feedback to the designers of the automated sentiment analysis to optimize the analysis’ future utilization potential. The results showed a fair automated-human agreement with rater 1 and rater 2 (k = 0.29) under categorical interpretation and a moderate automated-human agreement with rater 1 (ICC = 0.51) and rater 2 (ICC = 0.55) under continuous interpretation regarding the extraction of overall sentiment from the session records. The human-human agreement regarding overall sentiment was substantial under the categorical interpretation (k = 0.68) and good (ICC = 0.89) under the continuous interpretation. Furthermore, the automated analysis scored the sentiment of the session records more positive than the human raters. The automated analysis did not demonstrate increased difficulties in assessing sentiment related to specific types of EDs, despite its challenges with disorder-specific vocabulary.

### 4.1 Automated-human agreement

The findings of the automated-human agreement are partly in line with other studies. While this study found a moderate continuous automated-human agreement and a fair categorical agreement for both human raters, the exemplary study by Provoost et al. (37) found a moderate automated-human agreement under both continuous and categorical interpretations. Furthermore, a study investigating the performance of four different sentiment analyses compared to a baseline of multiple human raters found a fair automated-human agreement for three sentiment analyses and one moderate agreement, all under a categorical interpretation (38). Similarly, a study by Oksanen et al. (39) found a fair automated-human categorical agreement between an automated sentiment analysis and each of its three human raters, rating the sentiment of videos and comments related to AN.

Some shortcomings of the automated analysis could explain the findings of the automated-human agreement. The automated analysis’ lexicon did not include vocabulary of sentiment specific to an ED context and labeled negative words as positive and vice versa. Besides, the automated analysis assigned a sentiment score to words that were not sentiment-bearing and not considered by the human raters. Literature has shown that sentiment analyses are often domain and context-specific, accordingly, a word’s polarity may have been altered due to the context and domain within which it occurred and labeled as of the opposite sentiment (42–45). Furthermore, the automated analysis used “n-grams,” which only considered words before a sentiment-bearing word and not after; as a result, it may have overlooked the context of certain words and labeled them incorrectly. A study investigating the performance of different machine and deep learning methods showed that the accuracy of n-grams was best for unigrams (one-word sequences) and decreased with bigrams and trigrams, as these may contain more complex human language (61). These shortcomings could have led to a discrepancy in sentiment scores between the two analyses, leading to a lower automated-human agreement and potential more positive rating of the records’ sentiment opposed to the human raters.

Another explanation that may cause a variance in the sentiment scores between the two analyses is the difference in approach regarding the rating of the session records. The automated analysis’ word-by-word analysis with use of two and three-word combinations in comparison the human raters’ holistic interpretation of the records’ sentiment may result in diverging sentiment scores on the session records. This effect was amplified when only one or two words were rated by the automated analysis within a record compared to the human raters considering the entire record and, hence, caused a difference in the observed sentiment scores.

Furthermore, other possible explanations may be due to the characteristics of the session records. The records included occasional misspellings or incorrect sentences, implicit statements of sentiment, or varied in their length, content, and written language due to differences in writing of clinicians. This will make the extraction of sentiment from the records more complex and misinterpretation more likely by the automated analysis, whereas human raters possess the ability and intelligence to comprehend difficult and ambiguous sentences and to extract sentiment from these more precisely (40, 62). The automated sentiment analysis rated more records than the human raters due to its inability to consistently identify and exclude “irrelevant” records. This occasionally resulted in the algorithm rating records with minimal sentiment content, leading to outliers often paired with the human raters rating the records as “irrelevant.” Furthermore, the session records often contained a summary of patients’ difficulties and successes from the past days or weeks in between therapy sessions. Seventy percent of the session records classified as “neutral” within the human analysis were also categorized as “mixed,” meaning that the records contained both an equal positive and negative polarity. Furthermore, the automated analysis’ sentiment scores were mostly centered around zero, whereas the majority of the human raters’ sentiment scores were mostly centered around slightly positive or slightly negative, meaning that sentiment may be difficult to extract from session records, often containing sentiment from both polarities.

Furthermore, the sentiment within the session records does not directly stem from the patients; rather, it is a clinician’s interpretation of patients’ sentiment and may, therefore, contain a subjective view of clinicians. The human raters agreed to only score sentiment stemming from the patients. Whereas human raters are able to distinguish between sentiment stemming from the patient or the clinician, the automated analysis could not. The human raters were able to take this into account when scoring the records that could have resulted in the observed difference in sentiment ratings.

In summary, the automated analysis performed worse in discerning sentiment from session patient records as opposed to the human raters, meaning that the automated sentiment analysis cannot be used within practice in its current state, assuming that the gold standard of the human-human agreement is considered good enough.

### 4.2 Agreement between human raters

The finding of the substantial categorical human-human agreement is in line with previous research, which investigated the performance of an automated sentiment analysis against two or three human raters and found a substantial agreement as well, under the categorical interpretation (42, 63, 64). In contrast, a lower (moderate) categorical and continuous human-human agreement was found in the study of Provoost and colleagues (37), who used an average of eight human raters per text.

A possible explanation for the findings could be due to the human raters’ utilization of a feedback session and clear protocol. Likewise, a study by Moreno-Ortiz et al. (64) incorporated a feedback session to optimize the followed protocol. They found a significant increase in the human-human agreement between the first and second trial, ensuring that the session records were rated similarly. Furthermore, both raters of this study possessed knowledge of EDs as they were both educated within the field of psychology. Hence, they may have similarly interpreted words or expressions specific to an ED context and whether these were of positive or negative sentiment.

The human-human agreement within this study was chosen as the “gold standard” to compare the performance of the automated analysis with. Nevertheless, no perfect agreement has been found within literature regarding human-human agreement for the validation of an automated sentiment analysis within a mental healthcare context, meaning that human raters still lack consensus regarding the rating texts’ sentiment (42). For this reason, it cannot be determined with certainty whether the automated analysis performed either “good” or “bad” as there is no solid benchmark.

### 4.3 Qualitative differences between the automated sentiment analysis and human raters

The automated sentiment analysis was tailored to the Dutch language and mental healthcare context but not to the context of EDs. Hence, because of the context of the records and limited domain-specificity of the used lexicons, differences in positive and negative matches between the automated analysis and human raters were identified. Furthermore the automated analysis does not seem to encounter more difficulties with rating the sentiment within the context of a specific diagnosis, as each diagnosis once showed to have a lower automated-human agreement in comparison to the overall automated-human agreement, except for BN which showed a lower ICC value two times.

### 4.4 Strengths and limitations

A strength of this study is that the session records were written by trained clinicians providing real contextual data from patients from an actual ED treatment center. The findings of this study will also be provided as feedback to the developers of the automated sentiment analysis to improve its performance for future usage. Furthermore, the utilization of a feedback session may have supported that the records were rated similarly by the human raters (64). A limitation of this study was that there is no solid benchmark to compare the performance of the automated analysis with. The human raters were chosen as gold standard; however, the human raters still lack solid consensus when rating the session records. Hence, the results should be interpreted with caution. Furthermore, this study used fewer texts for the analysis than other research investigating the performance of automated sentiment analyses, as more than 40% of the records within this study were not suitable for the analysis, decreasing the reliability of the results and possibly leading to a selective sample of records (37, 39, 42, 65). The human raters only evaluated sentiment related to the patient, whereas the automated analysis rated an entire session record, which may have led to a discrepancy in the content evaluated by the human raters and the automated analysis. Therefore, the interpretation of the IRR between the human raters and the automated analysis requires caution. Another limitation is that the human raters may have been subjected to emotional bias, which is a distortion in one’s cognitions due to emotional factors such as personal feelings at the time of decision-making (66). Consequently, the affective state of the human raters at the time of rating the session records could have influenced the sentiment score that was given to a certain text. Furthermore, this study only included two human raters, which makes for a less representative interpretation of the overall sentiment within the session records compared to using multiple raters (67). Lastly, the method for the standardized sentiment scores regarding the category “neutral” differed between the automated analysis and human raters, as establishing a clear median or “neutral” point was challenging. The decision to use a range for the algorithm was made to accommodate the nuances and variability inherent in an automated sentiment analysis to represent the category “neutral.” However, this may have resulted in differences within the category “neutral” between the automated analysis and human raters.

### 4.5 Future research and implications

The findings suggest that the automated analysis performs worse than human raters in discerning sentiment from session records. However, it is questionable whether the human-human agreement can be considered the gold standard to determine the performance of the automated analysis. Nevertheless, no clinically relevant IRR values that would allow methods to be applied within practice could be identified within the literature sufficient enough to apply such methods within practice, and, therefore, although excellent reliability should be strived for, it is of interest to investigate what IRR values are sufficient enough to apply such methods within clinical practice.

This research is among the first to assess the performance of automated sentiment analysis on contextual patient data. Its potential application in clinical practice could serve as a feedback system, allowing for quick analysis of patients’ sentiment over time. This could be especially long-term treatments, where subtle changes in sentiment might be challenging to discern through manual review alone. Consequently, this approach could reduce the burden on both clinicians and patients and, importantly, aid in identifying when treatment adjustments are necessary or detect deterioration in patients’ conditions. Such an application could be a significant step forward in optimizing mental healthcare delivery.

For future research, it is recommended to increase the number of human raters and examine the differences between the raters’ sentiment scores in closer detail to improve the gold standard. Moreover, because of limited evidence regarding the utilization of human raters as the gold standard, patients’ ratings of their own moods after or before therapy sessions or utilization of patients’ diaries and accompanying mood ratings could make an additional benchmark to validate the automated sentiment analysis to. Furthermore, the sentiment scores of the automated analysis could be compared to therapists’ sentiment ratings of the session records, which may not only yield insightful information about the efficacy of the tool but also identify sentiments that might not be immediately apparent to the therapist and could give an additional layer of insight into patient progress.

Another key recommendation is to update the automated analysis lexicon with context-specific ED words and investigate its performance again on texts or session records within an ED treatment setting to improve its accuracy (68). Furthermore, potential confounding variables should be investigated by operating the automated sentiment analysis on more homogenized samples of texts with controlled participant demographics such as specific age groups and types of EDS to investigate the impact of different variables on the sentiment analysis.

Furthermore, the usability of session records for the extraction of patients’ sentiment can be questioned because of its characteristics and it is primarily an account by the clinician of the patients’ sentiment. Therefore, the sentiment of the session records and whether these could give an accurate representation of the patients’ sentiment should be further investigated. In addition, future research could focus on exploring novel procedures to document patients’ sentiment more directly, such as, by requesting the patient to summarize their feelings about the past week(s) in a few sentences at the beginning or end of a session, which could be used for the monitoring of patients’ sentiment over time.

However, despite the session records including complex and ambiguous information, which makes them difficult to analyze, the records do contain valuable information about processes and underlying patterns contributing to EDs. Hence, it may be particularly interesting to use an open coding, through which the session records are examined on recurring ED themes, which may be beneficial for the understanding of the mechanisms exhibited by individuals with an ED disorder. Furthermore, it would be particularly interesting to explore session records capturing both sentiment from patients and clinicians to investigate the therapeutic alliance and dynamic, as this is a contributing factor within treatment and may yield insightful information about such processes.

## 5 Conclusion

To conclude, this study suggests that the current automated sentiment analysis tool does not perform as well as human raters in discerning sentiment from session patient records within a Dutch ED treatment context when compared against the human-human agreement standard. However, it is crucial to acknowledge the limitations of this benchmark. The lack of a solid consensus among human raters on sentiment evaluation indicates a need for alternative benchmarks in future research to more accurately assess the efficacy of automated sentiment analysis tools in clinical practice, such as patients’ own mood ratings. Furthermore, this study showed that the sentiment of patients extracted from session records can be portrayed over time. Moreover, the automated sentiment analysis must be optimized by including context-specific ED terms and expressions within its lexicon to increase the analysis’ accuracy, requiring further investigation. Lastly, it remains uncertain whether the patient session records are suitable for the extraction of patients’ sentiments due to their complex and ambiguous nature containing both an equal number of positive and negative sentiment.

## Data availability statement

The raw data supporting the conclusion will be made available without undue reservation. However, the session patient records cannot be given without undue reservation due to the privacy of the participants. The patient session records of the participants can be made available upon reasonable request.

## Ethics statement

The studies involving humans were approved by Commission Ethics Psychology University of Twente. The studies were conducted in accordance with the local legislation and institutional requirements. The participants provided their written informed consent to participate in this study.

## Author contributions

SH: Data curation, Formal analysis, Investigation, Software, Writing – original draft. JK: Investigation, Software, Supervision, Validation, Visualization, Writing – review & editing. JdV: Conceptualization, Data curation, Formal analysis, Investigation, Methodology, Project administration, Resources, Software, Supervision, Validation, Writing – review & editing.
