# Supplementary material for: Examining a sentiment algorithm on session patient records in an eating disorder treatment setting: a preliminary study
Source: Front Psychiatry. 2024 Mar 13;15:1275236. doi: 10.3389/fpsyt.2024.1275236 (PMC10965787; doi:10.3389/fpsyt.2024.1275236)
Supplement: Supplementary file 1 [file Table_1.docx]

Supplementary Material

# Supplementary Data

**A. Consent form**

INFORMEDED CONSENT WETENSCHAPPELIJK ONDERZOEK

ONDERZOEK: HET TOEPASSEN VAN TEKST DATA ANALYSE OM INZICHT TE KRIJGEN IN DE BEHANDELVOORTGANG BIJ EETSTOORNISSEN

**Toelichting
Lees dit formulier alsjeblieft zorgvuldig. Als je anoniem gegevens van je behandeling beschikbaar wil stellen voor deze studie, dan kun je dat in dit formulier aangeven. De bijbehorende informatie brochure van het onderzoek kun je via deze link vinden. Hier kun je ook de contact gegevens van de onderzoekers vinden, mocht je vragen hebben over deelname aan deze studie.**

**Toestemmingsverklaring 1**

Met de ondertekening van dit document geef je aan dat je minstens 17 jaar oud bent; dat je goed bent geïnformeerd over het onderzoek, de manier waarop de onderzoeksgegevens worden verzameld, gebruikt en behandeld en welke eventuele risico’s je zou kunnen lopen door te participeren in dit onderzoek.

1. Ik kreeg voldoende informatie over dit onderzoeksproject. Het doel van mijn deelname in dit project is voor mij helder uitgelegd en ik weet wat dit voor mij betekent.

2. Mijn deelname in dit project is vrijwillig. Er is geen expliciete of impliciete dwang voor mij om aan dit onderzoek deel te nemen.

3. Mijn deelname houdt in dat gegevens die tijdens de behandeling verzameld worden gebruikt worden voor de doelen van het wetenschappelijke onderzoeksproject zoals beschreven in de informatiebrochure.

4. Het is mij duidelijk dat er bijzondere persoonsgegevens over mijn gezondheid en functioneren verwerkt worden en dat deze gegevens geanonimiseerd worden door de hoofdonderzoeker voordat er data-analyses plaatsvinden.

5. Het is mij duidelijk dat, als ik toch bezwaar heb met een of meer punten zoals hierboven benoemd, ik op elk moment mijn deelname, zonder opgaaf van reden, kan stoppen.

6. Ik heb van de hoofdonderzoeker de uitdrukkelijke garantie gekregen dat er voor wordt zorggedragen dat ik niet ben te identificeren in door het onderzoek naar buiten gebrachte gegevens, rapporten of artikelen. Mijn privacy is gewaarborgd als deelnemer aan dit onderzoek.

7. Ik ben akkoord met eventuele (wetenschappelijke) publicaties die voortkomen uit dit onderzoeksproject en ben mij ervan bewust dat een er een wettelijke bewaartermijn van de anonieme data van 10 jaar geldt na publicatie.

8. Ik heb de garantie gekregen dat dit onderzoeksproject is beoordeeld en goedgekeurd door de Commissie Ethiek Psychologie van de Universiteit van Twente onder registratienummer ….

9. Ik heb dit formulier gelezen en begrepen. Al mijn vragen zijn naar mijn tevredenheid beantwoord en ik ben vrijwillig akkoord met deelname aan dit onderzoek.

10. Ik ben tenminste 17 jaar oud.

Ik ben **akkoord/niet akkoord** met bovenstaande punten.

**Toestemmingsverklaring 2**

Ik geef toestemming om mijn data, welke geanominiseerd wordt gearchiveerd als onderdeel van het beschreven onderzoek, beschikbaar te stellen voor toekomstig onderzoek en lesdoeleinden door anderen onderzoekers en docenten. Ik begrijp dat deze gegevens nooit te herleiden zijn tot mij als individu.

Ik ben **akkoord/niet akkoord**.

**B. Information brochure**

<https://humanconcern.nl/wp-content/uploads/2022/05/Informatiebrochure-wetenschappelijk-onderzoek-2022.pdf>

**C. Protocol**

**Protocol for the human sentiment analysis**

In order for the human raters to assess the texts in an objective and similar way, a protocol was created to analyze the patient session record texts to attribute a sentiment score to each. Therefore, the following points must be considered when analyzing the texts:

1. Texts with less than five words will not be examined, nor will patient session records including information about work supervision, communication and arranged appointments with other clinicians or institutions and descriptions of actions taken by the clinician(s) regarding administrative activities.
2. Patient session record texts including phone calls, voicemails or mobile texts containing sentiment will be considered regarding sentiment.
3. *Results* written by the clinician about the patient's sentiment will be considered, as it contains sentiment from the patient.
4. When rating the patient session record, the diagnosis of the patient must be known and considered.
5. The examination and ratings of a patient session record text should be based, only, on the given patient session record text, previous texts belonging to the same patient and context outside a given text should not be considered when rating a certain text. However, metaphors and the meaning behind indirect sentiment will be considered.
6. The examinations and ratings of the patient session record texts should be based on the sentiment of the patient. Parts within the patient session record texts about the clinician's sentiment, subjective view, or treatment instructions (what the patient is going to do next) should not be considered.

**D. Wordlist with words specific to the Dutch contexts of EDs**

**Table 6.**

*Positive and negative context-specific sentiment words regarding ED from the human analysis*

|  | Sentiment |  |
| --- | --- | --- |
|  | Positive | Negative |
| General | Uitdagingen aangaan  Eet-uitdagingen  Herstel  Hulp vragen  Regie nemen  Open over emoties / emoties delen  Gezonde kant / gezonde gedachten  Verboden eetlijst proberen  Deelnemen aan het leven  Genieten (van eten)  Dankbaar(heid)  Minder regels  Behoeftes uitspreken / grenzen aangeven / voor jezelf kiezen  Gunnen  Eetstoornis op de achtergrond  Meer voelen  Trots op zichzelf  Angst loslaten  Kwartje gevallen  Emotionele lading minder  Besef  Luisteren hongergevoel  Toegeven emoties  Grip hebben  Aankomen  Openheid  met zichzelf in conact  dingen aangaan  minder wandelen / stappen zetten  bewustworden  groei doormaken  opgewekt  vrijheid  rust  kracht om tegen eetstoornis in te gaan  niet extra sporten  Normaal eten  geen paniek momenten  emotieregulatie  herstellijn  meedoen met anderen  flexibel zijn  gewich doet de patient niks | (Voor)compenseren / eten overslaan  Eetstoornis trekt  Eetstoornis nodig hebben  Eetstoornis opspelen/ last van eetstoornis  Eetstoornis is heftig / aanwezig’  Niet delen hoe het gaat/ onderdrukken van emoties /emoties niet delen  Braken/ braakgedachtes  Eetstoornis gedachtes  Schuldgevoel  Innerlijke criticus  Overspoeld door emoties  Niet meer in de hand  Vastzitten ‘eetstoornis-stem’  Regels  Eetbui / overeten  Overdenken  Afvallen  Veilige keuze  Zichzelf groot houden  Dik voelen / angst dik worden  Schaamte  Escape  Onrust  Negatieve lichaamsbeleving  Weinig eten  Niet luisteren naar grenzen  Restrictief eten  Niet bewust van honger gevoel / trek  Vreselijk om voor spiegel te staan  Kritisch op lijf  Eetbui-drang  Lijdensdruk  Terugval / vervallen oude patroon  Overleven  Uiterlijk controleren / controledrang  Afwezig contact  niet gezien voelen  prestatiedrang  bewijzen aan zichzelf  wandelen / stappen  obsessief |
| AN | Afbouwen sporten  Aankomen | Sporten (en elke vorm die daarbij komt kijken) |
| BN |  | Braken / braak gedachtes / overgeven  Eetbui / overeten |
| BED | Sporten  Afvallen | Eetbui / overeten |
| OSFED | Afbouw sporten |  |
|  |  |  |

**E. Sentiment words categorized by the automated sentiment analysis as of positive or negative polarity but not within the human analysis**

**Table 7**

*Sentiment words categorized by the automated analysis as a positive or negative match which were not within the human analysis*

| **Positive** | **negative** |
| --- | --- |
| Sporten  Compenseren  Controle  Bewegen  Bekend  Waarneming  Baan  Reis  Bekend  Soort  Beleving  Buiten  Vakantie  Aanpassen  Kenmerken  Definitief  Informatie  Rest  Kind  Vorm | Lichaamsbeleving  Emotieregulatie  Geur  Adhd  Instelling  Te ervaren  Systeem  Geen therapie  Klein  Te bereiden  Vet  Klinische  Kwetsbaar  Brood  Kistje |
